# Mediated Generalization and Stimulus Equivalence

**DOI:** 10.1007/s40614-021-00281-3

**Published:** 2021-02-24

**Authors:** Christoffer Eilifsen, Erik Arntzen

**Affiliations:** Department of Behavioral Sciences, Oslo Metropolitan University, P.O. Box 4, St. Olavs plass, 0130 Oslo, Norway

**Keywords:** Mediated generalization, Mediated associations, Stimulus equivalence, Derived responding, History

## Abstract

From the 1930s to the 1970s a large number of experimental studies on mediated generalization were published, and this research tradition provided an important context for early research on stimulus equivalence. Mediated generalization and stimulus equivalence have several characteristics in common, notably that both traditions seek to experimentally investigate derived responding among arbitrarily related stimuli in human participants. Although studies of stimulus equivalence are currently being regularly published, few studies investigate mediated generalization in humans today, and the research tradition is mainly of historical interest. The current article will give an account of the origin, the development, and the demise of research on mediated generalization, including a presentation of publication trends, experimental methodology, and the conceptual context research on mediated generalization took place within. Finally, some thoughts on the demise of mediated generalization and its relevance for modern research on stimulus equivalence and other types of derived responding are presented, including reflections on the observability of explanatory variables and the use of inferential statistics.

In the contemporary experimental analysis of behavior processes in humans, a major topic of investigation is the study of stimulus equivalence. An active field of inquiry since the early 1970s, research on stimulus equivalence involves establishing responding in line with arbitrary relations among stimuli through operant reinforcement procedures. Subsequently, tests for patterns of responding among these stimuli that are related to this training, but have not themselves been trained, are administered under extinction conditions. If a systematic pattern of responding having the properties of reflexivity, symmetry, and transitivity is observed, responding in accordance with stimulus equivalence is said to be present (Sidman & Tailby, 1982). For example, if a set of stimuli A and a set of stimuli B are related through reinforcement, followed by the same set of stimuli B being related to another set of stimuli C, symmetry is evident if responding show B stimuli to be related to A stimuli and C stimuli to be related to B stimuli. Transitivity is considered present if responding showing A stimuli to be related to C stimuli is evident. Reflexivity is the matching of identical stimuli involved in the training but is not commonly tested for in human participants.

When Sidman introduced and started developing a research program on stimulus equivalence in 1970s and 1980s, this was done in the context of other traditions studying derived responding among arbitrarily related stimuli (Sidman, 1971; Sidman & Tailby, 1982). One such tradition that was the study of mediated generalization or mediated associations (Earhard & Mandler, 1965; Jenkins, 1963; Kjeldergaard, 1968), and research from this tradition is commonly cited in early papers on stimulus equivalence (Lazar, 1977; Sidman & Cresson, 1973; Sidman, Cresson, & Willson-Morris, 1974). We will consider the terms “mediated generalization” and “mediated associations” as synonyms in the current article but employ “mediated generalization.” Horton and Kjeldergaard (1961, p. 2) define mediated generalization as the transfer of stimulus control “across different physical dimensions [relying] on response produced cues, developed through the learning process, as mediating links.” Earhard and Mandler (1965, p. 347) use the term “mediated associations” and define it as an “association [that] can be established *indirectly* between two verbal units by associating each unit independently with a third. The third verbal unit is then said to constitute a mediating link which serves to bridge the two original units.” In light of these two definitions, we offer our own generic definition of mediated generalization using more modern terminology: mediated generalization is an improvement or change in responding in the presence of a stimulus because of a learning history establishing an arbitrary relation between this stimulus and another stimulus or response that allows the latter to function as a commonly unobserved mediator that cause the change or improvement in behavior. Unlike stimulus equivalence, defined as relations between stimuli only, mediated generalization typically involves both stimuli and responses. For example, after establishing relations between stimuli in a set A and responses in a set B, followed by the establishment of relations between stimuli in the same set B and responses in a set C, mediated generalization will be considered present if this leads to an improvement in the learning of relations between stimulus set A and response set C due to the mediation of stimuli and/or responses of set B.

Modern stimulus equivalence research and studies of mediated generalization have several characteristics in common. First, both research traditions take place within scientific frameworks that, although they have important differences, are variations of behaviorism. Furthermore, research on mediated generalization was exclusively conducted with human participants, and, although there are studies of stimulus equivalence using nonhuman animals, humans are usually the participants in experiments on stimulus equivalence as well (Dube, McIlvane, Callahan, & Stoddard, 1993). In addition, both research on stimulus equivalence and studies of mediated generalization investigate derived responding among arbitrarily related stimuli. In the current context, the term “arbitrarily related” refers to a relationship among stimuli based solely on convention and not on physical similarity, whereas “derived responding” refers to responding related to previous learning that reliably occurs even though the behavior in question has never been explicitly trained, established, or reinforced. Finally, in both the literature on mediated generalization and stimulus equivalence it has been argued that research within these traditions illuminate behavior related to language or verbal behavior, as such behavior also involves arbitrarily related stimuli and derived responding (e.g., Jenkins, 1965b; Place, 1995/1996).

In the past decades, a research tradition distinct from, but related to stimulus equivalence called relational frame theory (RFT) has emerged, occasionally presented as studying  nonequivalence relations (Cooper, Heron, & Heward, 2020; Critchfield, Barnes-Holmes, & Dougher, 2018). Although research under the RFT umbrella concerns itself with stimulus equivalence, patterns of derived responding among stimuli arbitrarily related in other ways than by equivalence are also studied. Such relations, referred to as frames, may for example be opposition (e.g., Whelan & Barnes-Holmes, 2004), comparison (e.g., Vitale, Barnes-Holmes, Barnes-Holmes, & Campbell, 2008), or hierarchy (e.g., Stewart, Slattery, Chambers, & Dymond, 2018). Stimulus equivalence, within RFT called the frame of coordination, is considered just one of many frames (Hughes & Barnes-Holmes, 2016). Sidman refers to stimulus equivalence responding as emergent (Sidman, 1994), whereas authors in the RFT tradition typically refer to patterns of previously unreinforced behavior as derived (Hayes, 1996). We will throughout this article use “derived responding” as an umbrella term referring to several different kinds of response patterns that are untrained or unreinforced, including responding in accordance with stimulus equivalence, performance indicating mediated generalization, and responding in line with relational frames other than stimulus equivalence. Because of the direct historical connection between stimulus equivalence and mediated generalization, the current article will focus on this relationship. However, discussions on mediated generalization and stimulus equivalence in the current article will also have relevance for research in the RFT tradition that is concerned with derived responding among arbitrarily related stimuli.

The most extensive treatment of mediated generalization in the context of modern research on derived responding to date appears in Sidman’s *Equivalence Relations and Behavior: A Research* Story (Sidman, 1994). Here, presentations and discussions of various topics involving mediated generalization are scattered throughout the book and used to underscore conceptual and methodological points relevant to the development of Sidman’s research program on stimulus equivalence. Discussions on and presentations of mediated generalization also appear in the literature on stimulus equivalence and other derived responding in a variety of settings both in the years before (e.g., Barnes & Holmes, 1991; Fields, Verhave, & Fath, 1984; Green, Mackay, McIlvane, Saunders, & Soraci, 1990; Wasserman & DeVolder, 1993) and the decade following Sidman’s (1994) treatment (e.g., Green & Saunders, 1998; Horne & Lowe, 1996; Mandell, 1997; Zentall, 1998), as well as in more recent literature (e.g., Critchfield et al., 2018; Critchfield & Twyman, 2014; Urcuioli, 2013). Although numerous, these appearances are brief and embedded in works mainly concerned with other topics. As such, an article providing an in-depth presentation of mediated generalization in the context of modern research on derived responding that extends the work of Sidman (1994) and others is warranted.

The current article will present mediated generalization by giving an overview of the history of this research tradition. This has several purposes. To look at history may provide a trajectory of the events leading to a contemporary research tradition, and as such say something about the current field. This endeavor may also point toward practices that have previously failed to sustain a research tradition, and this can potentially serve as warnings for the contemporary field (Coleman, 1995). The current article will therefore trace the development of the study of mediated generalization from its beginnings in the 1930s through its peak in the 1960s and subsequent decline and disappearance in the 1970s and 1980s. This will be done by presenting the experimental methodology used to investigate mediated generalization, publication trends, and the conceptual context in which the research took place. Following this we will offer some thoughts on the possible relevance of the fate of research on mediated generalization for contemporary studies of derived responding.

## Classical Conditioning Procedures and the Shipley-Lumsdaine Paradigm

In 1935 Walter C. Shipley published an empirical article on what he termed indirect conditioning that would be highly influential for research on mediated generalization. In this article Shipley employed the terminology and procedures of classical or respondent conditioning, with behavior being conceptualized as elicited and experimental preparations set up to establish conditioned responding through the pairing of stimuli. The target study of the paper, by Shipley called Group A, is illustrated in the upper left portion of Table 1. In this study, male college students were exposed to a procedure establishing a wink of the eye as a conditioned response to a buzzer sound and a flashing light. This was done by pairing these two initially neutral stimuli to the unconditioned stimulus of striking the cheek of the participant with a small metal rod. The flash was subsequently paired with a mild electric shock to the participant’s finger, the latter being an unconditioned stimulus eliciting finger withdrawal. Following these stimulus pairings, the flash elicited both the conditioned response winking and conditioned response withdrawal of the finger, whereas the buzz elicited winking. Shipley then investigated whether finger withdrawal occurred in the presence of the buzz, a stimulus that had never been paired with the shock. Six of the 10 participants withdrew their finger upon presentation of the buzz. Shipley labeled the phenomenon indirect conditioning, as he assumed the buzz elicited the withdrawal response not because of a learning history directly pairing it to an unconditioned stimulus, but because of its relationship to another stimulus, the flash, that had been paired with an unconditioned stimulus, the shock.

**Table 1:** Group A and Group C in Shipley (1935) illustrated with and without unobserved mediating variables

	Procedure			Mediated Generalization Interpretation				
	CS	US	R	CS	US	[Mediating R]	[Mediating S]	R
Group A								
Phase 1	Buzz	Strike	Wink	Buzz	Strike	**―**	**―**	Wink
Phase 2	Flash	Strike	Wink	Flash	Strike	**―**	**―**	Wink
Phase 3	Flash	Shock	Withdrawal	Flash	Shock	[Wink]	[Wink]	Withdrawal
Test	Buzz	**―**	Withdrawal	Buzz	**―**	[Wink]	[Wink]	Withdrawal
Group C								
Phase 1	Buzz	Strike	Wink	Buzz	Strike	**―**	**―**	Wink
Phase 2	**―**	Strike	Wink	**―**	Strike	**―**	**―**	Wink
Phase 3	Flash	Shock	Withdrawal	Flash	Shock	[Wink]	[Wink]	Withdrawal
Test	Buzz	**―**	Withdrawal	Buzz	**―**	[Wink]	[Wink]	Withdrawal

The left side of the table shows the procedure and results, whereas the right side shows the procedure and results with the unobserved mediating variables shown in brackets. *CS* conditioned stimulus, *US* unconditioned stimulus, *R* response, *S* stimulus

At first, Shipley did not consider the results of Group A particularly puzzling, nor did he conclude that the results were difficult to explain using the theoretical framework he worked within. He proposed that the derived responding observed when the buzz elicited the finger withdrawal occurred because (a) two stimuli (flash and buzz) had entered the same class when acquiring the same function (wink), and (b) that a function (finger withdrawal) later established to one member of such a stimulus class resulted in similar stimulus control spreading to other members of this class. This explanation is virtually identical to the definitions of functional or acquired equivalence in the contemporary literature on stimulus control (Dougher & Markham, 1994; Urcuioli, 2006). Following the successful production of indirect conditioning in Group A, Shipley went on to study the behavior of other participants in several control conditions. These control conditions resulted in few participants responding in a manner indicative of indirect conditioning, but one condition proved to be an exception. In this condition, called Group C and illustrated in the bottom left portion of Table 1, a strike to the cheek was paired with a buzz, followed by a pairing of a flash with a shock. To make the procedure as similar to Group A as possible, it also included presentations of the strike alone without pairing it another stimulus. The procedure resulted, as expected, in the conditioned response wink being elicited by the buzz and the conditioned response finger withdrawal being elicited by the flash. As for Group A, finger withdrawal was then assessed when presenting the buzz. Contrary to Shipley's expectations, 7 of the 10 participants withdrew their finger when the buzz was presented. This finding could not be explained by functional equivalence, because the buzz and the flash was never established as members of the same stimulus class.

To explain the results of Group C, Shipley proposed that the shock elicited not only finger withdrawal but also an unobserved wink as an unconditioned response. In addition, inspired by Hull’s thinking on the behavioral effects of kinesthetic or proprioceptive stimulation (Hull, 1931), Shipley suggested that the wink as a response was also a stimulus capable of eliciting further responding and came to mediate responding in presence of the buzz. The conditioning procedure and resulting responses, including the invoked mediating wink, are illustrated in the bottom right portion of Table 1. According to Shipley then, the presentation of the shock in the flash–shock pairing resulted in an unobserved wink occurring as a response. As the unobserved wink was also considered a kinsethetic stimulus, its pairing to the shock also gave it the function of eliciting finger withdrawal through classical conditioning. The later buzz-strike pairing resulted in, as expected, the wink being elicited by the buzz, but once it occurred, the wink would then as a stimulus elicit finger withdrawal. In sum, the finger-withdrawal in the presence of the buzz was explained by Shipley as mediated by an unobserved event, the wink.

Shipley then went on to reinterpret the results of Group A using the mediating wink as an explanatory variable. This interpretation is illustrated in the right upper portion of Table 1. As described above, the procedure for Group A involved the pairing of both a flash and a buzz to a strike, resulting in the elicitation of a wink in the presence of both stimuli. This was followed by the pairing of a flash to a shock, resulting in finger withdrawal in the presence of the flash. Derived responding was then observed when the buzz elicited finger withdrawal. According to Shipley’s reinterpretation the shock in the flash–shock pairing did, as in Group C, elicit an unobserved wink as an unconditioned response to the shock. When, in the test for derived responding, the buzz elicited the wink due to its previous pairing with the strike, the wink as a stimulus further elicited the finger withdrawal response. Again the finger-withdrawal in the presence of the buzz was explained as mediated by the unobserved wink.

As the mediation interpretation could explain both the derived responding of participants in Group A and in Group C, Shipley concluded that this explanation had greater generality than one based on functional equivalence and should be preferred. Unlike an explanation based on functional equivalence, however, this explanation included a mediating variable that was unobserved. A replication of Shipley’s study by Lumsdaine (1939), however, reported observations of winks during the flash–shock pairing and derived responding. Lumsdaine’s study, however, was only reported as an abstract from the proceedings of a conference, so the actual data appear to be lost. Regardless, the experimental set-up and the proposed mediation mechanism reported in Shipley (1935) and Lumsdaine (1939) became known as the Shipley-Lumsdaine paradigm.

## Mediated Generalization and Paired Associate Procedures

The 1930s and 1940s saw a few infrequent publications on phenomena related to mediated generalization (Cofer & Foley, 1942; Peters, 1935). By the early 1950s, however, several research groups working within various strains of behaviorism picked up the Shipley-Lumsdaine paradigm, and the number of publications on the topic increased. As Shipley, these researchers conceptualized behavior as elicited by stimuli, but the language and procedures of classical conditioning were no longer used. Studies did not focus on the pairing of stimuli and the effect this had on elicited responses, but rather emphasized the pairing of or association between stimuli and responses. In addition, stimuli and responses involved in experiments were now mostly verbal, considerably different from the stimuli and responses in Shipley (1935) and from those typically associated with classical conditioning procedures in general.

Although there were exceptions (e.g., Grice & Davis, 1958; Jeffrey, 1953), by far the most common experimental preparation used to study mediated generalization from the 1950s and onward was the paired associate procedure. This procedure involved training participants to respond to lists of stimuli, with different responses being assigned to different stimuli in these lists. The stimuli and responses were typically nonsense syllables, although meaningful words were also commonly used. The relationship between the stimuli and the responses was usually arbitrary in the sense that there was no physical similarity relating the stimulus and the response in each pair. To study mediated generalization in such preparations, at least two interrelated lists of stimulus–response (S–R) pairs must be trained. For example, in a study by Bugelski and Scharlock (1952), lists that each contained 16 nonsense syllables were presented to 20 college students with the goal of establishing S–R pairs. Each nonsense syllable was presented separately. First, the presentation of one of the nonsense syllable from what was called List A was, after a time gap, followed by the presentation of the syllable from List B the experimenter had assigned to be paired with this particular syllable. Participants were instructed to read the B syllable and eventually say it out loud *before* it appeared as a stimulus. Trials relating specific stimuli from list A to specific stimuli from List B were randomly presented to the participants. Once the participant responded by saying the experimenter-defined correct B syllable out loud in the presence of the other half of the pair from the A list five times in a row the, an A–B relation was considered mastered. No operant reinforcement was arranged and A syllables as stimuli were thought of as eliciting specific B syllables as responses once the mastery criterion was reached. Another set of to-be-learned S–R associations were subsequently introduced. Now, syllables from the B list served as the potentially eliciting stimuli, whereas another set of syllables, the C list, was the potential responses. Again, participants were instructed to respond to the second syllable by reading it out loud and eventually to say it out loud before it appeared in writing, with the goal of pairing specific stimuli form list B to specific responses in list C. Once all B–C associations were considered mastered using the five correct trials in a row criterion, a test for mediated generalization was administered. In this test, List A syllables served as the potentially eliciting stimuli and the List C syllables served as the responses, A syllable from List A was presented to the participant, followed by a syllable from List C, and the participant was once again instructed to read the C syllable and eventually pronounce it before it appeared in writing. Unlike the previous two lists, the learning of the A–C syllable pairs was thought to potentially be mediated by the connection the A stimuli and C responses had to list B syllables, because the latter set of syllables previously appeared both as elicited responses in the A–B training and eliciting stimuli in the B–C training.

**Fig. 1:**
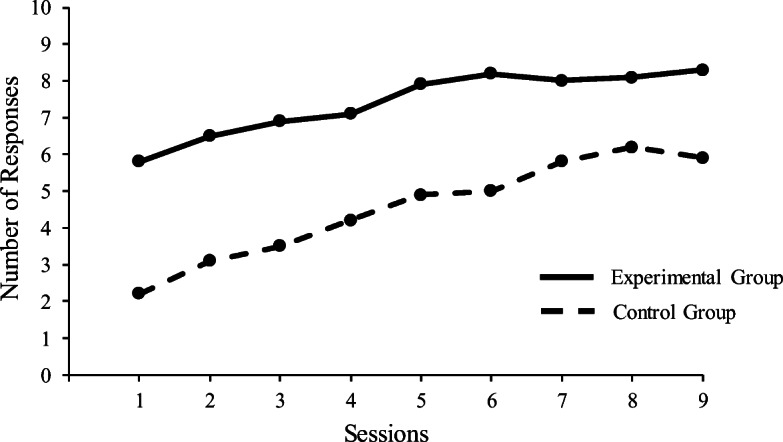
Hypothetical data illustrating the savings method commonly used to measure mediated generalization. *Note.* The figure depicts, in a similar manner to illustrations occasionally seen in the mediated generalization literature, the average number of correct responses across participants per session of 10 trials in an experimental condition consisting of A–B, B–C training, and an A–C test (solid line) and a control group consisting of A–B, C–D training, and an A–D test (broken line). This average difference in correct responding over sessions between the experimental group and the control group is referred to as “savings in learning” and was considered the dependent variable indicating or showing mediated generalization. See the text for further details

Mediated generalization as a dependent variable was measured in the Bugelski and Scharlock (1952) study by using a variation of the savings method (Hebb, 1958, p. 246). To illustrate this way of measuring performance, Fig. 1 shows hypothetical, but fairly typical, data obtained in studies of mediated generalization. The figure shows the difference between the average responding of experimental group participants in an A–C test following A–B, B–C training and the average responding of control group participants in an A–D test following A–B, C–D training. Note that in contrast to the A–C test in the experimental condition, there is no possibility of a learned indirect connection between the stimuli and responses for the A–D associations of the control condition test. The figure shows that participants in the experimental group on average respond correctly, that is, say the experimenter-defined correct syllable out loud before it appears in writing, on more A–C trials compared to the responding of the control group on A–D trials, throughout nine tests sessions. Like many other authors writing about mediated generalization, Bugelski and Scharlock (1952) assumed that savings or quicker learning for the experimental group occurred because the presentation of A syllables elicited unobserved B syllables as responses, which as unobserved stimuli further elicited the observed C responses in the experimental condition, a chain of events similar to the explanation involving a mediating wink proposed in the Shipley-Lumsdaine paradigm.

In the Bugelski and Scharlock (1952) study, participants served as their own control by learning both experimental and control lists, but this later became a rarity. By far the most common way of studying mediated generalization in the following decades, was to compare average performance in experimental and control groups using the savings method by analyzing these data using inferential statistics and significance testing, typically by conducting *t*-tests or analysis of variance (ANOVA). If significant differences between the experimental and control groups were obtained using such analyses, mediated generalization was thought to have occurred.

One possible way to connect the mediated generalization tradition to modern research on stimulus equivalence is to invoke Sidman’s (2000) theory of stimulus equivalence and the role this theory has for responses in generating derived responding. In this theory, Sidman proposes that all elements of an operant reinforcement contingency enter into a stimulus equivalence class, including the response. The behavior seen in paired associate mediated generalization experiments were not thought of as operant by the researchers in this tradition, however, and experiments on mediated generalization did not include any events that were meant to functions as operant reinforcers in the procedure. Leaving aside this and the fact that the defining properties of stimulus equivalence were not fully assessed in tests for mediated generalization, it is possible envision an explanation of the derived performance seen in mediated generalization tests as relying on responses becoming part of stimulus equivalence classes. In a typical paired associate mediated generalization experiment, this would involve that all stimuli and responses of a mediated generalization stimulus–response chain become members of a stimulus equivalence class. For example, learning a stimulus A–response B relation, and then a stimulus B–response C relation would lead to an ABC stimulus equivalence class, presumably explaining the derived performance on AC tests for mediated generalization without having to appeal to mediating variables. However, it is far from clear that a stimulus equivalence class was established in the data typically obtained in mediated generalization tests. If a stimulus equivalence class had been established in these tests, one would assume that this would lead to relatively errorless performance, especially after some of the C stimuli had been presented to the participants. Errorless performance, or anything close to it, is typically not seen in these tests, where the participants’ performance, although significantly different from a control group, included many errors over several sessions. See Fig. 1 for an illustration of typical performance of groups of individuals. There are probably several reasons for this. The large number of small stimulus classes typically involved in mediated generalization experiments, a relatively low number of repetitions of relations during the training procedure, the lack of programmed consequences, and the fact that the participants had to produce responses vocally, may all be variables that affected behavior. Modern studies on acquired equivalence using matching-to-sample procedures in nonhuman animals may be of relevance for shedding light on these issues and may more generally be a viable way of studying performance similar to mediated generalization without committing to the theoretical and methodological restraints of this research program as it is presented the current article. See, for example Urcuioli (2006), for an overview of such modern research on response-based derived performance in nonhuman animals.

## Mediated Generalization Publication Trends

**Fig. 2:**
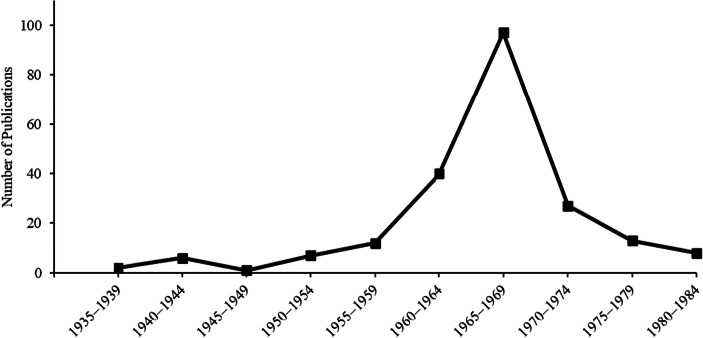
The number of publications on mediated generalization between 1935 and 1984. *Note.* The figure shows the number of returned results when searching for mediat* generalization, mediat* associat*, or mediat* paradigm* in the PsychINFO database. The results are organized in 5-year blocks from 1935 to 1984

Figure 2 shows the number of publications that appear when searching for truncated versions of the terms mediated generalization, mediated association, and mediation paradigm in the PsychInfo database in the 50 years between 1935 to 1984 in 5-year blocks. Following a few publications on the topic in the 1930s and 1940s, the number of publications started to rise throughout the 1950s. As the 1950s turned into the 1960s, the number of publications on mediated generalization increased dramatically, eventually reaching around a hundred publications in the latter 5 years of the 1960s. In the 1970s, however, the number of publications declined from what in hindsight was a peak in the number of publications. This happened quickly, so that the first 5 years of the 1970s saw fewer publication than the first 5 years of the 1960s. By the 1980s, the number of publications was fewer still, and the number drop to a level not seen since the 1940s. By the 1980s, many papers that appear when searching for mediated generalization and similar terms in the PsychInfo database, no longer include any mention of paired associate procedures or references to the preceding literature on mediated generalization (e.g., Boyd & Levis, 1983; Charlop, 1983). As such, it seems like these terms now have been given new content assumingly unrelated to how they were used in the past. Taking this into consideration, one may argue that the number of publications on mediated generalization is virtually zero by 1980.

## Other Research Traditions Relevant to Mediated Generalization

Two fields of inquiry that partly overlap with and at times influence the study of mediated generalization is the study of semantic generalization and the study of mediated transfer. On occasion, studies of sematic generalization, mediated transfer, and mediated generalization are treated as part of the same research program in modern literature in the topic (e.g., Fields et al., 1984; Sidman, 1994). Indeed, the use of the terms was not consistent in the literature from 1930 to 1980 either and sometimes the three terms are used interchangeably. Because of this, we find it necessary to define and present semantic generalization and mediated transfer and relate these terms to the study of mediated generalization.

Before presenting semantic generalization, it is, however, useful to first clear up the relationship between the terms mediated generalization and stimulus generalization. Stimulus generalization refers to the behavior process where stimuli that are physically similar to, but different from those present when reinforcement or learning occurred, come to exert control over behavior (Catania, 2013). Although both mediated generalization and stimulus generalization have the word generalization in their names, we argue that these behavior processes are quite different. For instance, although stimulus generalization involves responding under control of dimensions related to a single stimulus involved in the prerequisite training, mediated generalization is a more complex process involving derived performance based on training with several stimuli. The use of the word generalization in mediated generalization may have come about because the term historically also has been used to describe experiments where classical conditioning procedures involving verbal materials as conditioned stimuli also lead to the conditioned response being elicited by synonyms of these verbal materials (Cofer & Foley, 1942). For example, Riess (1940), using college students as participants, paired a loud noise with 50 common words, including the words urn and surf. Galvanic skin responses (GSR) to homonyms such as earn and serf and synonyms such as vase and wave were then then measured. A GSR measure larger than preconditioning was detected for both homonyms and synonyms. Although GSR responding to the homonyms can be explained by respondent generalization, GSR responding to the synonyms seem to require something more to be explained. Researchers suggested that the responses were conditioned not just to words as stimuli, but also to the meaning of words. The meaning of a word was then thought to be linked to other verbal stimuli such as synonyms, thus explaining “generalization” involving synonymous bearing no physical resemblance to the training stimuli (Razran, 1939; Riess, 1940). This phenomenon was also called semantic generalization as the proliferation of the effects of conditioning seemed similar to the behavior process of stimulus generalization, although in the case of semantic generalization this spread was not related to the physical dimensions of stimuli, but to their sematic meaning. Some researchers proposed that this occurred because, unobserved to the experimenter, the presented stimuli elicited covert synonyms that subsequently elicited the observable response. Although studies of semantic generalization did not necessarily set forth to study mediated generalization, some researchers assumed that the underlying process was similar, with some suggesting that the paired associate mediated generalization procedure was merely an intraexperimental demonstration of semantic generalization (Jeffrey & Kaplan, 1957; Maltzman, 1968).

Studies of mediated transfer, on the other hand, were conducted in order to experimentally investigate the cumulative effects of different episodes of learning. Transfer was called mediated when the experimental procedures and results called for the inclusion of unobserved variables that mediated observed responding (Barnes & Underwood, 1959). The tradition overlaps with studies on mediated generalization, but is broader, studying both the facilitation and interference of previously trained relations on the learning of new relations using a variety of procedures, some quite different from the ones used to study mediated generalization (e.g., Kausler & Dean, 1967; Kendler, 1972; Richardson, 1967). For example, interference through transfer was considered evident if the teaching of A–C relations in a paired associate procedure negatively affected performance on tests of previously learned A–B relations, as both B and C stimuli were now connected to the same A stimuli and the C associations disturbed responding when B responses were requested. Mediated generalization can be viewed as a special case of the *facilitation* of performance through mediated transfer. The mediated transfer tradition would influence research on mediated generalization in the 1960s, and we will give an example of this influence when later discussing the phenomena of pseudomediation.

## The Conceptual Context of Research on Mediated Generalization

Lumsdaine’s (1939) attempt to gather empirical evidence for the mediating variable suggested by Shipley’s 1935 study was not followed up by later researchers. In contrast, many authors placed the mediating variable at a level beyond behavior and apparently beyond potential observability. The following comment on the Shipley-Lumsdaine paradigm from Osgood’s (1953) influential textbook *Method and Theory in Experimental Psychology* is illustrative:

There were some cases, however, in which the withdrawal reaction antedated the eyelid movement, and this suggests that the winking movement may be only an *overt index of the actual mediation process*. This is what would be expected according to the mediation hypothesis: in the original training, the light (sign) was presumably becoming associated with the fractional anticipatory portions of the reaction to tap-on-cheek (stimulus-object), and it is this mediation process which is more or less faithfully indexed by the overt winking (p. 462; emphasis added).

Although exceptions eventually appeared (e.g., Yarmey & Csapo, 1968), no focus was initially put on establishing or teaching mediated generalization to individuals not responding in such a manner. This left the burden of explaining derived responding solely on the current environment and the unobserved mediating variable. In order to explore why researchers of this period found it appropriate to leave the crucial mediating variable unobserved and outside the boundaries of experimental manipulation in this manner, it is useful to describe some of the conceptual commitments of these researchers and discuss how they differ from the conceptual framework of behavior analysis today.

Sidman’s modern research program on stimulus equivalence arose in the context of behavior analysis, a science of behavior associated with the philosophy of radical behaviorism. Characteristics of behavior analysis include an emphasis on operant behavior, a goal of prediction and control of the behavior of individuals, and empirical foundations consisting of a relatively large proportion of experimental studies on the behavior of nonhuman animals. Neither behavior analysis nor radical behaviorism is much discussed in the literature on mediated generalization. The few instances we have been able to locate include two references to Skinner’s (1957) book *Verbal Behavior*, where similarities between Skinner’s ideas about private control of behavior and mediated generalization are mentioned (Horton & Kjeldergaard, 1961; Maltzman, 1968). No empirical studies typically associated with behavior analysis were to our knowledge ever cited in the mediated generalization literature. As such, studies of mediated generalization can be said to have taken place in research traditions in experimental psychology that were largely disconnected from behavior analysis.

The traditions mediated generalization took place within can all be characterized as forms of behaviorism. These experimental traditions have been labeled methodological behaviorism, neobehaviorism, associationistic psychology, and S–R psychology, among other things (Maltzman, 1968; Mandler, 1962), the names reflecting changes over time in these forms of behaviorism, but also differences among them. A common thread in all these research traditions was, however, that the major scientific unit of analysis was considered to be the relationship between preceding stimuli and subsequent responses. Many of the conceptual developments in these fields were related to accommodating incoming experimental data to this S–R unit. Changes in research on mediated generalization reflect wider trends in these forms of behaviorism from the 1930s to the 1970s, culminating in the emergence of theoretical commitments associated with cognitive psychology. Some authors have characterized theses conceptual changes as a gradual development (Leahey, 1992; Moore, 2011/2012), an historical analysis that stands in contrast to the abrupt and radical changes suggested by the term “cognitive revolution” (Miller, 2003). Two types of theoretical variables, intervening variables and hypothetical constructs, play a central role in this historical analysis, and exploring these terms further is of interest in order to illuminate the role of the mediating variables in mediated generalization.

In his 1913 behaviorist manifesto, John B. Watson proposed that the S–R relation would be a suitable unit of analysis in developing field of behaviorism, and later, inspired by the works of Pavlov and Bekhterev, expanded on this statement by declaring the conditioned reflex to be a central mechanism of learning (Watson 1916, 1925). Shipley (1935) is a good example of a study in this S–R tradition, and Shipley’s article does indeed cite both Pavlov and other Russian researchers studying reflexes.Various challenges to such a “pure” S–R behaviorism quickly arose. The derived performance seen in the described Group C in Shipley (1935) can be considered an example of experimental results that are difficult to account for by using S–R units alone, because there are no eliciting stimuli in the immediate environment that seem to explain the difference in responding observed for participants in the experimental condition compared to the control conditions. To solve such problems, the strict S–R focus was abandoned, and it became common to include theoretical terms, later called “intervening variables,” in the analysis of behavior. Research traditions that employ intervening variables are sometimes referred to as S-O-R neobehaviorism, with the O being the contribution of the organism toward explaining the relationship between the S and the R. A concern of many researchers at the time was how experimental psychology could remain a proper empirical science while at the same time using such organismic intervening variables as a part of scientific practice. A solution was to attempt treat intervening variables strictly as devices for generating theory and facilitating communication among researchers. Intervening variables were conceptualized as not having an ontological status other that being descriptions of experimental manipulations and their relation to experimental results. In other words, they were not considered to be observable nor to have a role in causing behavior (MacCorquodale & Meehl, 1948; Moore, 2011). Examples of terms often conceptualized as intervening variables include the term “habit strength” as used by Hull (1943) and the term “purpose” as used by Tolman (1925). Both Hull and Tolman were influential proponents of expanding the classical S–R behaviorism to include intervening variables (Kimble, 1994).

The proprioceptive variable Shipley (1935) used to illuminate the derived responding seen in his experimental data, especially as further explored in the previous quote from Osgood (1953), may be viewed as an intervening variable. Seen as an intervening variable, the proprioceptive variable is not thought to have an existence but is rather a purely theoretical concept invoked to communicate the relationship between the independent and dependent variables in Shipley’s experiment. Shipley (1935) talked about mediated generalization using terms such as “stimuli” and “responses,” a vocabulary also used to describe observable events in the environment. Other authors writing about mediated generalization used a terminology different from the stimuli and responses of the environment to denote the mediating variable, with the variable making derived responding possible being referred to as an associative link (Palermo, 1962), visualization (Murdock, 1952), covert verbalization (Norcross & Spiker, 1958), mediating symbolic processes (Russell & Storms, 1955), and associative arousal (James & Hakes, 1965).

The mediating variables invoked by Shipley (1935) and other researchers do not, however, always seem to fit the definition of intervening variables as mere summaries of experimental manipulations. In many instances they seem to be referring to events that potentially can be observed and have independent causal effects on the responding of the participants. For example, James and Hakes, James, and Lloyd (1965) attribute the failure to show mediated generalization as being due to the “lack of associative arousal” (p. 89). Many of these terms seem more like hypothetical constructs, unobserved theoretical variables inferred from observable events that have a casual role in explaining observable behavior. Unlike intervening variables, hypothetical constructs are thought to have an existence and to be potentially observable (Borsboom, Mellenbergh, & van Heerden, 2003; MacCorquodale & Meehl, 1948).

To distinguish between intervening variables and hypothetical constructs is, however, often difficult, and this is also the case in research on mediated generalization. One may argue that intervening variables exclusively defined in terms of operations in the environment are in practice rare, and that most theoretical terms conceptualized as intervening variables actually consist of surplus meaning with explanatory power beyond stimuli manipulated by the experimenter (Leahey, 1980). In hindsight, it may be more reasonable to conceptualize the theoretical variables used in research on mediated generalization as hypothetical constructs, but hypothetical constructs that varied in complexity and described with various degree of detachment from the vocabulary used to describe the stimuli and responses of the environment. During the decades of research on mediated generalization, there was a tendency for these hypothetical constructs to become more complex and less related to the vocabulary of stimuli and responses, although such development was by no means universal among researchers and writers publishing on the topic. We will return to the subject matter of hypothetical constructs after presenting some issues debated in the mediated generalization literature that proved difficult to resolve.

## Unresolved Issues in the Mediated Generalization Tradition

Studies of mediated generalization included the manipulation of many different independent variables, such as the use of stimuli of different modalities (Shapiro & Palermo, 1968), varying the meaningfulness of stimuli (Aagard & Stone, 1972), changing the amount and type of exposure to the trained relations (Peterson, 1964), and using stimuli from word association test norms with varying degrees of associability (Russell & Storms, 1955). Some studies that may be of interest to modern researchers on stimulus equivalence include studies on the effect of instructions (e.g., Lee & Jensen, 1968; Yarmey & Csapo, 1968) and the use of mediated generalization procedures to teach concept formation (e.g., Petre, 1964; Yarmey, 1969). Many studies were, however, mainly concerned with exploring details of the unobserved mediating variable, a topic we will argue the mediated generalization tradition was particularly ill-suited to illuminate. Indeed, prior to the decline in the number of published studies on mediated generalization, researchers faced several findings that generated theoretical debates that proved difficult to resolve. Two such findings were the lack of mediated generalization in so-called four-stage paradigms and uncertainties related to the phenomena of backward association and pseudomediation.

Sidman (1994) spends several pages discussing the challenges faced by researchers of mediated generalization when studying so-called four-stage paradigms and we will briefly review the issue here. When there were two sets of associated lists to be learned in a paired associate mediated generalization experiment, it was commonly called a three-stage paradigm, as it consisted of two training stages (i.e. A–B and C–B), and one test stage (i.e. A–C). Under many circumstances mediated generalization was shown to occur in three-stage paradigms. However, some researchers attempted to study mediated generalization in four-stage paradigms involving an additional list of stimuli. For example, Jenkins (1963) trained A–B, B–C, A–D relations followed by a C–D test for mediated generalization. It proved, to the surprise of many researchers, difficult to secure performance in line with mediated generalization in this and other four-stage paradigms. Commonly invoked theories in the mediated generalization literature involving the association among stimuli and responses in a partly unobservable chain did not anticipate that there would be a large discrepancy between yields in the three- and four-stage paradigms. Some authors suggested that this apparent lack of mediation was caused by the unlearning or extinction of relations trained early in the procedure (James & Hakes, 1965; see also Sidman, 1994). For example, in Jenkins’s (1963) procedure described above, the training of A–D relations at a later stage of the experimental procedure possibly extinguished previously trained A–B relations and therefore broke the chain of associations necessary to show mediated generalization. Neither the theoretical frameworks invoked to explain mediated generalization, nor the procedures used for studying mediated generalization were, however, geared toward observing the mediating variables or disturbances to them. Because of the explanation based on extinction remained speculative and only a trickle of experiments attempted to illuminate the issue further (Grover, Horton, & Cunningham, 1967; Hakes et al., 1965; James & Hakes, 1965).

Another debate in the mediated generalization literature was concerned with the role of backward association in what was called pseudomediation. Backward association refers to the influence of relations among stimuli and/or responses on behavior in the opposite direction to how those relations have been trained. Backward association is related to the term symmetry in Sidman and Tailby’s (1982) definition of stimulus equivalence, but there is a key difference between the two terms. Although symmetry is concerned with relations among stimuli, backward association may include relations among both stimuli and responses. In several of the experimental preparations studying mediated generalization, it was assumed that backward association was taking place and that this event was partly responsible for the savings in learning seen in tests for mediated generalization. For example, in one experimental preparation lists of A–B and then C–B relations were trained, and mediated generalization was tested in A–C test trials (Jenkins, 1963). Savings that occurred were explained by mediated generalization that involved the unobserved backward association of B–C. That is, nonsense syllables or other stimuli that that figured as the responses of the participant during training, came to exert influence on behavior as stimuli during the test. Backward association was rarely directly assessed in experiments investigating mediated generalization, but commonly seen as a given and a necessary prerequisite for mediated generalization (Jenkins, 1963). This was, however, disputed by other researchers (Battig & Koppenaal, 1965), with others again settling for a middle ground where backward association was seen as consistently being established, but in a weaker form than the trained forward associations (Horton & Kjeldergaard, 1961). The issue of backward association became involved in a debate on so-called pseudomediation, which called much of the theory associated with mediated generalization into question. When A–B and B–C lists were trained, followed by the training of A–C relations, the common assumption was that savings on A–C learning was due to A stimuli eliciting unobserved B responses, which as stimuli, again unobserved, elicited observed C responses. Some researchers associated with the mediated transfer tradition describe above, offered an alternative explanation that did not regard performance as related to such mediation at all (Mandler & Earhard, 1964). In this explanation, it was proposed that the training of B–C relations lead to the unlearning of the previously trained A–B relations due to inference through backward association. That is, learning of the B–C relations caused the participants to unlearn the A–B relations, as B stimuli were now associated with C stimuli and not A stimuli. This then, caused the participants to learn the A–C relations quicker in the test for mediated generalization, as the B responses did not get in the way responding in line with the A–C relations. Control groups, for example involving A–B, C–D training, and A–D tests, never experienced the unlearning of A–B relations, and therefore learned the A–D relations slower. Mandler and Earhard (1964) provided data in support of the phenomenon. The validity and generalizability of the experiment was, however, contested by other researchers (Horton, 1967; Jenkins, 1965a). The experiments of the mediated generalization tradition were, however, ill-suited for investigating whether the performance seen in mediated generalization experiments were the result of mediation or backward association and unlearning, because the events crucial to either explanation were unobserved in these experiments. As such, the issue of pseudomediation proved difficult to resolve.

## Mediated Generalization and Hypothetical Constructs

In the 1960s, several researchers studying paired associate learning, including mediated generalization, started proposing more complex mediating mechanisms to explain data that was obtained. The change in the types of theoretical variables employed took place in reaction to the restrictions of the intervening variables and stimulus–response associations that had dominated as theoretical variables in experimental studies of paired associate learning and mediated generalization (Mandler, 1962; Palermo, 1971). For example, Battig (1968) argued that current research findings “clearly cannot be accounted for on the basis of presently available S-R principles” (p. 164) and proposed several new learning mechanisms responsible for observable behavior. Battig and other researchers now talked about paired associate learning, including mediated generalization, as a problem-solving process and invoked such terms and ideas as the identification and classification of stimuli and responses, the encoding of stimuli, the hierarchical availability of associations, recall processes, and linguistic or logical rules (Battig, 1968; Earhard & Mandler, 1965; McGehee & Schulz, 1961). For example, Mandler (1962) explains the performance-enhancing effects of overtraining S–R relations in paired associate procedures as follows:

Analogic structures permit covert trial and error, i.e., cognitive manipulation of previously established behavior. In this sense, the analogic representation of a prior behavior sequence is one possible “hypothesis” to be applied to a particular situation. Given many such structures which are relevant to a situational input, the several structures will occur seriatim and covertly until an appropriate one is expressed behaviorally. . . . Structures are developed on the basis of associationist stimulus-response relationships but, once established, enable the organism to behave “cognitively.” (pp. 417–418)

Although some previously proposed intervening variables in paired associate learning and mediated generalization seemed to leave part the explanation for responding somewhere other than in the environment or the behavior of the individual, many researchers now explicitly left part, if not most of the burden, of explaining performance on mediating mechanisms. These proposed structures seem like clear-cut examples of hypothetical constructs with surplus meaning described in a vocabulary indicating that they differ from observable stimuli or responses (MacCorquodale & Meehl, 1948). Many found that these hypothetical constructs provided satisfactory explanations of mediated generalization and resolved debates on backward association, pseudomediation, and the lack of mediated generalization seen in the four-stage paradigm (Battig, 1968; Earhard & Mandler, 1965). The preference for hypothetical constructs was, however, by no means universal among researchers, and whether explanations for mediated generalization should be based on stimulus–response associations or hypothetical or cognitive structures were commonly debated in the literature (Goss, 1961; Jenkins, 1963). This development did not, however, lead to any increased interest in conducting research on mediated generalization. On the contrary, as it became more fashionable to employ hypothetical constructs as explanations for behavior in the 1960s and as cognitivism came to dominate experimental psychology in the 1970s, the number of research papers on mediated generalization declined. Although the term “mediated generalization” occasionally appears in contemporary empirical studies of derived responding in nonhuman animals (e.g., Nakagawa, 2005; Urcuioli & Lionello-DeNolf, 2005), mediated generalization as a field studying human behavior has lain dormant since the early 1980s. Research on mediated generalization was apparently unable to separate from its roots in S–R based forms of behaviorism. Several researchers that studied mediated generalization went on to have distinguished careers in other fields, such as cognitive psychology (Mandler, 1985) and mental chronometry (Jensen, 2006), but their latter-day careers did not involve research on mediated generalization.

## Methodological Differences in Research on Stimulus Equivalence and Mediated Generalization

Although both stimulus equivalence and mediated generalization are research traditions that set forth to experimentally investigate derived responding in humans, the two traditions employ different methods for doing so. Table 2 gives an overview of what we believe are important distinctions in procedures used to study mediated generalization and stimulus equivalence. The table shows differences for typical procedures, and the literature on mediated generalization as well as stimulus equivalence include studies where other procedures are used to train and/or test mediated generalization and stimulus equivalence. In contrast with the previously described paired associate learning used in the study of mediated generalization, research on stimulus equivalence commonly involves matching-to-sample (MTS) procedures being used both to train the stimulus relations that are the prerequisite for stimulus equivalence as well as testing for stimulus equivalence. Such procedures involve a generic selection response, such as pointing or responding to stimuli by using a computer mouse. MTS procedures are considered operant, so when establishing the prerequisite relations for stimulus equivalence, responses are followed by consequences indicating whether responding is correct or incorrect, whereas stimulus equivalence is tested in extinction. The establishment of the prerequisite relations and stimulus equivalence is commonly measured by assessing the precent responses that are in line with these trained relations and in accordance with the defining properties of stimulus equivalence (reflexivity, symmetry, and transitivity).

**Table 2:** Overview of the typical elements of procedures for training prerequisite responding and testing for mediated generalization and stimulus equivalence

Experimental procedure	Mediated generalization	Stimulus equivalence
Training and test of derived responding	Stimulus–response pairing	Matching-to-sample
Training consequences	None	Operant reinforcement
Test consequences	None	Extinction
Theoretical variable of interest	Unobserved mediator	Response pattern (Relextivity, Symmetry, Transitivity)
Measurement of dependent variables in test	Trials to criterion	Percent correct
Data reported as	Experimental group average, relative to a control group average (Savings)	Individual performance

Please note the more direct assessment of stimulus equivalence responding compared to the savings method used to measure mediated generalization. The paired associate learning procedure and the indirect assessment of mediated generalization apparently encouraged the development of theory invoking unobserved variables to explain derived responding. That directly observable patterns of responding are considered the central dependent variables in research on stimulus equivalence presumably makes it less pressing to include unobserved variables in the explanation of such derived responding. Indeed, several theoretical accounts that propose to explain stimulus equivalence responding do not include mediating variables (e.g., Hughes & Barnes-Holmes, 2016; Sidman, 2000).^1^ Furthermore, some of the debates that plagued research on mediated generalization have been resolved on empirical grounds in modern stimulus equivalence research, presumably because trained and derived responding is available for observation. For example, derived responding has been repeatedly shown to occur with more than one—and occasionally, several—nodes separating stimuli in a class, and environmental variables affecting such responding can be, and have been specified (Arntzen, Grondahl, & Eilifsen, 2010; Fields, Adams, Verhave, & Newman, 1990; Fields & Moss, 2007). As such, the use of MTS to study stimulus equivalence has made it possible to empirically investigate derived performance in four-stage paradigms, an achievement never attained in the mediated generalization tradition. In general, creating a way to study derived responding where the responding of interest is observed directly is probably one of the most important achievements of Sidman’s modern conceptualization of stimulus equivalence and this is likely to have played a central role in the continued viability of research on stimulus equivalence and RFT over several decades.

## Mediated Generalization and Modern Research on Derived Relations

In a recent article on Sidman and contemporary research on stimulus equivalence, Critchfield et al. (2018) state that “Sidman also knew that the existing research on mediated generalization had not reaped many dividends, perhaps because of its association with models like Hull’s that were top-heavy with hypothetical constructs” (p. 16). We interpret this to mean that the authors suggest that Sidman’s modern conceptualization of stimulus equivalence was formulated in light of previously active research traditions that did not remain productive over time because of their commitment to conducting research in order to assess Hullian models. We partly agree with this suggestion, but we believe the sentiment provided by the quote needs to be modified and extended to give a more accurate description of mediated generalization and the relationship between mediated generalization and modern research on stimulus equivalence.

First, the mediated generalization research tradition was not particularly “heavy” with complex hypothetical constructs for most of its history. Rather, many, if not most, researchers studying mediated generalization relied on the relatively simple idea of associations between stimuli and responses, albeit with some of these associations being unobserved. Hull is occasionally cited in the mediated generalization literature, but these citations typically refer to Hull’s early contribution of distinguishing between primary and secondary generalization, with the latter term occasionally being used synonymously with mediated generalization (Horton & Kjeldergaard, 1961; Hull, 1939). The complex mathematical formulations describing intervening variables commonly associated with Hull (e.g., Hull, 1943) were never a part of the mediated generalization research tradition. We argue instead that issues went unresolved and there was a lack of sufficient dividends in the mediated generalization tradition because the crucial explanatory variables went unobserved, not necessarily because of their complexity or association with Hullian models.

Second, we argue that although the unobservability of the explanatory variable is likely to be one of the reasons for the decline in research on mediated generalization, there are probably other factors influencing this decline. Another distinct difference between research on mediated generalization and modern stimulus equivalence research is the way derived responding is measured and analyzed. Sidman’s development of stimulus equivalence relied on data from studies focusing on the effects of environmental variables on the behavior of individuals (Sidman, 1994). Research on mediated generalization, on the other hand, almost exclusively used inferential statistics comparing the behavior of groups in order to make conclusion of the effects of independent variables on mediated generalization. The latter practice may lead to several problems for a research program that are well-known to behavior analysts. First, excessive use of inferential statistics analyzing the behavior of groups decreases the ability of researchers to observe the functional relations between experimental manipulations and the behavior of individuals that is traditionally the topic of investigation in behavior analysis and the basis for predicting and influencing behavior in this tradition. In addition, basing conclusions about the effects of independent variables solely on group inferential statistics increases the probability of concluding that that independent variables have an effect on dependent variables, when they if fact do not. This practice may then encourage continued research on “synthetic” phenomena, with little value in driving forward a research program aimed at predicting and influencing behavior (Baer, 1977; Blampied, 2013; Sidman, 1960). So, in addition to the extensive use of unobserved explanatory variables, the group statistical inference approach may have played a role in impeding the understanding of mediated generalization and in generating and continuing unresolved debates, such as the described debates on performance in four-stage paradigms, on backward association, and on pseudomediation. For example, the discussion around pseudomediation was based on a few experiments on relatively small groups of participants. The previously mentioned study by Mandler and Earhard (1964) included 44 college student participants evenly divided into an experimental group that received training and testing predicted to generate performance in line with pseudomediation and a control group that received training and testing predicted not to lead to such performance. Results were reported as a visual display showing average trials to criterion performance for the two groups and an ANOVA involving this measure and average performance in other stages of the experiment for the two groups. As was the case in virtually all studies on mediated generalization from the mid-1950s onward, information about the responding of individual participants was not reported. This way to report the data from experiments in the mediated generalization tradition makes it is difficult to say anything about individual performance, and in the Mandler and Earhard (1964) experiment, whether the proposed mechanism of pseudomediation is influencing behavior of individual participants or not. Nevertheless, Mandler and Earhard’s (1964) article generated much debate (Horton, 1967; Jenkins, 1965a; Postman, 1971) and some effort to conduct experiments on the topic of pseudomedition (Goulet & Postman, 1966; Kausler & Deichmann, 1968; Schulz, Weaver, & Ginsberg, 1965).

Thus, the fate of mediated generalization should not be dismissed as solely the result of the use of hypothetical constructs from a bygone era. The demise of the research tradition can also be considered a result of the excessive use of practices that exist in the contemporary literature on derived responding in behavior analysis today. Although it is currently the exception, rather than the norm, it is not uncommon to see studies in the modern literature on derived responding that present results of experiments exclusively as the analysis of the behavior of groups using inferential statistics, omitting information about the behavior of individual participants (e.g., Amd, de Oliveira, Passarelli, Balog, & de Rose, 2018; Bordieri, Kellum, Wilson, & Whiteman, 2016; Plazas & Villamil, 2016). In some preparations studying derived responding, notably implicit relational assessment procedures (IRAP), data analysis is virtually always conducted on the behavior of groups of participants, and data on the behavior of individual participants are seldom reported (e.g., Leech & Barnes-Holmes, 2020). Of course, there is nothing inherently problematic with the use of group inferential statics in behavior analysis. However, an excessive reliance on this type of data analysis can lead researchers into blind alleys and unproductive lines of research such as those we have argued was part of the demise of research on mediated generalization.

## Concluding Remarks

The history of mediated generalization illustrates that there was considerable interest in derived responding in experimental psychology and behaviorism for many years prior to Sidman’s introduction of the research program on stimulus equivalence. Research on mediated generalization, however, differs from modern research on stimulus equivalence and related types of derived responding in important ways and this seems at least partly related to the conceptual context within which the research took place. Whereas studies on mediated generalization were conducted in the context of various forms of behaviorism focusing on S–R relations, modern research on derived responding is related to behavior analysis and radical behaviorism. In the former, immediate antecedents of derived responding were studied in groups of participants, and unobserved causes took on a prominent role as explanatory devices. By contrast, researchers in the behavior analytic tradition will typically invoke the effect of past consequences for responding as part of an explanation of behavior, adhere to an insistent monism and anti-mentalism leading to a disregard for unobservable explanatory variables, and employ research methods geared toward uncovering relations between the environment and the behavior of individuals (Moore, 1999; Skinner, 1984). Working within the latter tradition, Sidman generated a new approach to an old area of interest that increased the accessibility of explanatory variables for individual behavior, solving some of the problems that had plagued the study of mediated generalization.

The investigation of stimulus equivalence has currently been an active area of research for approximately as long as the entire lifespan of research on mediated generalization and has led to the development of other ways of studying derived relations, in particular the RFT tradition. Scrutiny should, however, continually be applied to ensure that the current conceptualization of stimulus equivalence and other forms of derived responding promote research programs that reap dividends that are as large as possible. One purpose of presenting the history of mediated generalization has been to stir up discussions on conceptual and methodological issues relevant for the contemporary study of derived responding, such as debates on the consequences of using different units of analysis and various ways to measure and analyze central concepts. We believe that such discussions will influence the future development of research on derived relations, both in the context of behavior analysis and in the wider framework of behavior science.

## Footnotes

1 Some modern accounts of stimulus equivalence do include mediating behavior as part of explanation of stimulus equivalence, most prominently the naming account of stimulus equivalence (Horne & Lowe, 1996). Although naming may reasonably be called a mediation account and in this sense is similar to mediated generalization, naming also differs from mediated generalization in important ways. Perhaps most important, the theoretical development of and research on naming takes place in an operant tradition and is concerned with uncovering and establishing the learning history responsible for generating the behavior involved in naming. This is in contrast to the mediated generalization tradition, where the concern was with stimuli immediately preceding derived responding and the unobserved variables mediating this stimulus–response relation. The aim of this article is to present the history of mediating generalization, which is *a* mediation account of derived responding. Considering mediation accounts of derived responding *in general* is, however, beyond the scope of the current article.
